# Syndromic gastrointestinal stromal tumors

**DOI:** 10.1186/s13053-016-0055-4

**Published:** 2016-07-19

**Authors:** Riccardo Ricci

**Affiliations:** Department of Pathology, Università Cattolica del S. Cuore, Largo Agostino Gemelli, 8, I-00168 Rome, Italy

**Keywords:** Syndromic GIST, Hereditary GIST, Familial GIST, PDGFRA-mutant syndrome, Carney’s triad, Carney-Stratakis syndrome, Succinate dehydrogenase, germline mutations

## Abstract

Gastrointestinal stromal tumors (GISTs) are the most common mesenchymal neoplasms of gastrointestinal tract. They feature heterogeneous triggering mechanisms, implying relevant clinical differences. The vast majority of GISTs are sporadic tumors. Rarely, however, GIST-prone syndromes occur, mostly depending on heritable GIST predisposing molecular defects involving the entire organism. These conditions need to be properly identified in order to plan appropriate diagnostic, prognostic and therapeutic procedures.

Clinically, GIST-prone syndromes must be thought of whenever GISTs are multiple and/or associated with accompanying signs peculiar to the background tumorigenic trigger, either in single individuals or in kindreds. Moreover, syndromic GISTs, individually considered, tend to show distinctive features depending on the underlying condition. When applicable, genotyping is usually confirmatory.

In GIST-prone conditions, the prognostic features of each GIST, defined according to the criteria routinely applied to sporadic GISTs, combine with the characters proper to the background syndromes, defining peculiar clinical settings which challenge physicians to undertake complex decisions. The latter concern preventive therapy and single tumor therapy, implying possible surgical and molecularly targeted options.

In the absence of specific comprehensive guidelines, this review will highlight the traits characteristic of GIST-predisposing syndromes, with particular emphasis on diagnostic, prognostic and therapeutic implications, which can help the clinical management of these rare diseases.

## Background

Gastrointestinal (GI) stromal tumors (GISTs) are the most common GI mesenchymal neoplasms [1, 2]. They generally express KIT (CD117) and DOG1, similar to interstitial cells of Cajal (ICC) [3, 4].

GISTs have been a paradigm of molecular targeted therapy since they revealed activating *KIT* mutations [5], leading to the successful employment of the tyrosin kinase (TK) inhibitor (TKI) imatinib [6]. Since then, besides *KIT* mutations (~¾ of cases), GISTs have revealed other possible triggers: *platelet-derived growth factor (PDGF) receptor α* (*PDGFRA*) mutations (~7 %), succinate dehydrogenase (SDH) complex deficiency (~5 %, half of which depending on mutations of *SDH* subunits), and mutations of *BRAF* (2 %) or *neurofibromatosis type 1* (*NF1*) (1, 5 %). This heterogeneity implies different pathogenic, diagnostic and prognostic features characterizing distinct GIST subgroups, entailing diverse therapeutic approaches [1].

All of these pathogenic mechanisms but *BRAF* mutations have been rarely described to involve the entire organism, causing syndromes featuring multiple GISTs and peculiar associated signs. The resulting repertoire of syndromic GISTs constitute ~3-4 % of GISTs. Within them, NF1-associated GISTs prevail [1]. Much rarer are GISTs hinging upon germline *KIT* or *PDGFRA* mutations, with <50 kindreds/individuals described [7–48].

An overall favorable prognosis has been attributed to GISTs when multiple (including syndromic ones), no matter their number/phenotype [27]. However, despite the relatively high fraction of indolent GISTs due to NF1 or SDH-deficiency, “multiple GISTs” is a crucible where heterogeneous conditions merge in, differing in pathogenesis, prognosis and therapy. The approach to syndromic GISTs must therefore be personalized, considering the characters of both individual tumors, influencing their own natural history, and of the background syndrome, defining peculiar clinical settings.

GIST-predisposing syndromes will be herein reviewed, emphasizing inherent diagnostic, prognostic and therapeutic implications. Additionally, pertinent fundamentals of GIST pathogenesis will be recalled.

### Features of GIST-predisposing syndromes

#### *KIT* mutant syndrome

**Fig. 1:**
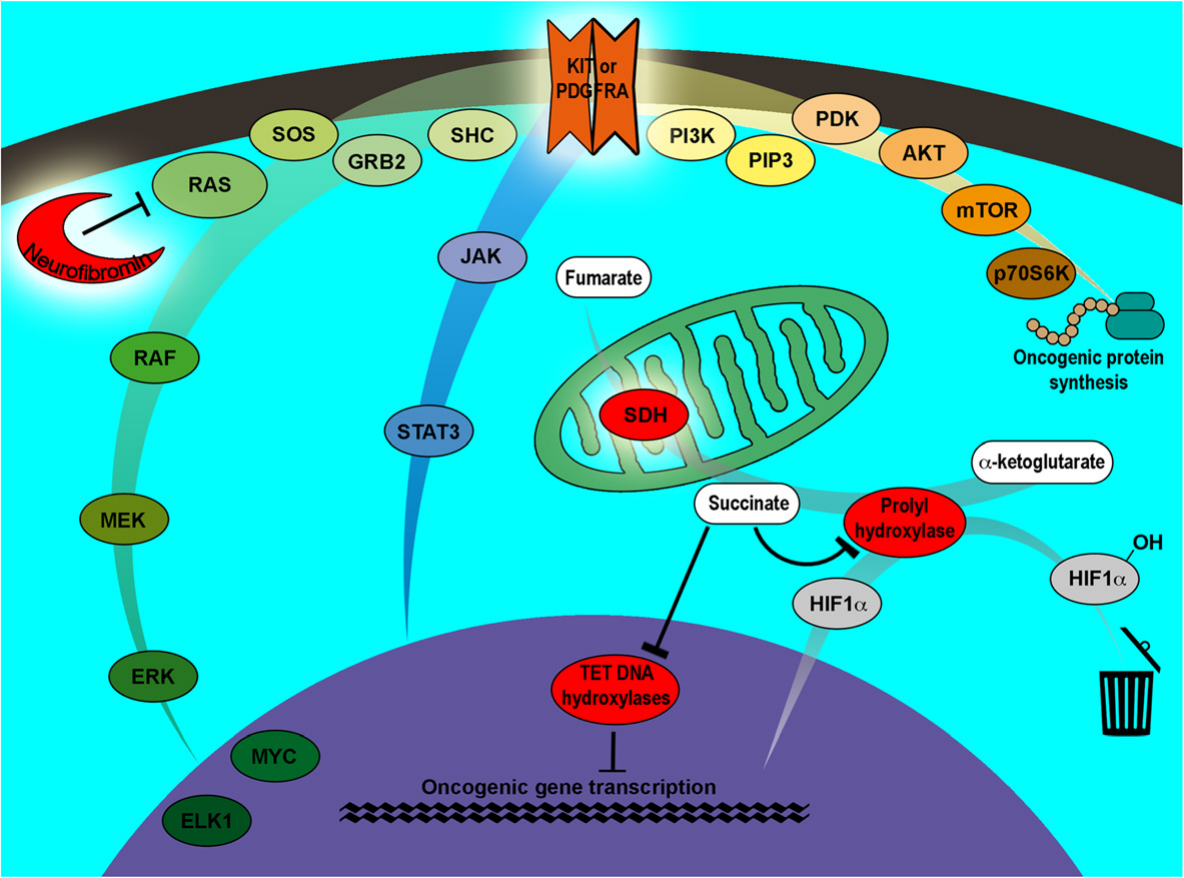
Molecular triggers and intracellular pathways involved in syndromic GIST arousal. Syndromic GISTs reported so far hinge upon alterations of one of the following (evidenced with a halo): KIT, PDGFRA, neurofibromin or SDH. KIT and PDGFRA activation initiates a downstream signaling involving multiple pathways: RAS/RAF/MEK/ERK (MAPK) (left, green hue); JAK/STAT3 (centre, blue hue); and PI3K/AKT/mTOR (top right, yellow/brown hue), stimulating oncogenic gene transcription or protein synthesis. In NF1-associated GISTs, tumoral inactivation of the WT *neurofibromin* impairs its RAS inhibiting effect, resulting in the activation of MAPK cascade downstream to KIT and PDGFRA. Impairment of the SDH enzymatic complex prevents succinate conversion to fumarate. Accumulated succinate inhibits prolyl-hydroxylase; the missed hydroxylation of HIF1-α prevents the degradation of this molecule which, consequently, heterodimerizes with HIF1-β and translocates into the nucleus acting as an oncogenic transcription factor. Furthermore, succinate accumulation inhibits TET DNA hydroxylases resulting in impaired conversion of 5-methylcytosine to 5-hydroxymethylcytosine, required for DNA demethylation, thereby influencing gene expression

KIT is a transmembrane type III TK receptor (TKR), whose gene is mapped to 4q12. Physiologically, KIT activation follows homodimerization upon stem cell factor (SCF) binding. Mutated KIT homodimerizes in a ligand-independent way. Activated KIT initiates RAS/MAPK, PI3K/AKT/mTOR and JAK/STAT3 signaling [49–51] (Fig. 1).

At the best of my knowledge, germline *KIT*-mutant GISTs have been described in 31 kindreds and 6 individuals without familial history [7–44]. Additionally, ungenotyped kindreds with signs coherent with *KIT*-dependent familial GISTs have been described prior to the discovery of *KIT* role in GISTs [52–55].

Average age-at-diagnosis of germline *KIT*-dependent GISTs anticipates that of sporadic cases of ~10 years: late forties/early fifties versus early/middle sixties [20, 29, 56–58]. This is true also if, in case of *KIT*-mutant kindreds, only the first individuals diagnosed with GIST (or GIST-compatible tumors in “pre-KIT era”) are considered, resulting in a 48-year mean, not influenced by familial screening. This anticipation likely parallels the early GIST trigger intrinsic to the connatal *KIT* mutation. The probability of GIST diagnosis in germline *KIT*-mutants increases with age, raising from 0.077 before the age of 40 to 0.462 by the age of 50 [29]. The age-at-diagnosis decrease reported in successive generations [17] is possibly due to subclinical GISTs diagnosed at screening.

*KIT*-mutant syndrome features no sex predilection, as expected given its autosomal dominant inheritance. Penetrance for GISTs is high [13, 20], unlike that for altered skin pigmentation [29].

Familial *KIT*-mutant GISTs occur along the whole GI tract (especially in small bowel/stomach), featuring a spindle, epithelioid or spindle-and-epithelioid citology (with the former prevailing), commonly expressing CD117 and DOG1 (Table 1), similar to their sporadic counterparts.

**Table 1:** Kindreds and individuals affected by gastrointestinal stromal tumors associated with germline *KIT* mutations

Reference	Type of report (family vs. single individual)	Mutant exon (mutation)	GIST			Other manifestations					
			Site^a^	Histology^b^	M^c^	ICCH^d^	Altered skin pigmentation	Mast cell disorders	GI motility disorders	Diverticula	Others
Nishida et al. [7]	family	11 (p.V560del)	SI^e^	S, M^f^			Perineal hyperpigmentation				
O’Brien et al. [8]; Hirota et al. [9]; Chen et al. [10]	family	11 (p.W557R)	SI	S, M	yes	yes					
Isozaki et al. [11]; Handra-Luca et al. [12]; Bachet et al. [13]	family	13 (p.K642E)	ST, SI	S, M	yes	yes	Lentigines on trunk, limbs, palms and soles		Dysphagia		
Maeyama et al. [14]	family	11 (p.V559A)	ST, SI	S			Hyperpigmentation and nevi				
Beghini et al. [15]	family	11 (p.V559A)	ST, SI	S, E, M	yes	yes	Hyperpigmentation of face, trunk, extremities and mucous membranes	Urticaria pigmentosa			
Hirota et al. [16]	family	17 (p.D820Y)	ST, SI	M^f^		yes			Dysphagia		
Robson et al. [17]	family	11 (p.W557R)	ST, SI	S, M		yes	Hyperpigmentation of hands, knees, perineum and circumoral areas		Dysphagia	Small bowel	
Antonescu et al. [18]	individual	11 (p.W557R)	ST, SI	S		yes					
Carballo et al. [19]	family	11 (p.L756_P577InsQL)	ST, SI	S		yes	Hyperpigmentation of neck, hands, feet and circumoral area				
Li et al. [20]	family	11 (p.V559A)	ST, SI	S^g^		yes	Hyperpigmentation, lentigines, café-au-lait macules^h^, nevi (all these lesions variably involved neck, perioral area, scrotal region/pelvic/genital/inguinal area, axillae, buttocks); vitiligo	Urticaria pigmentosa			1 Melanoma, 1 angioleiomyoma of ankle skin
Tarn et al. [21]	family	11 (p.D579del)	ST	M	yes						
Hartmann et al. [22]	family	8 (p.D419del)	SI	M		yes		Mastocytosis	Dysphagia		
Kim et al. [23]	individual	11 (p.V559A)	SI	S, M		yes					
O’Riain et al. [24]	family	17 (p.D820Y)	ST, SI, ICV	M^f^, S	yes	yes			Dysphagia	Small bowel	
Miettinen et al. [25]; Lasota and Miettinen [26]	family	11 (p.D579del)	SI, A, C	NS							
Kang et al. [27]	family	11 (p.V560G)	SI	NS		yes	Hyperpigmentation				
Kang et al. [27]	individual	11 (p.V559A)	SI	NS		yes					
Graham et al. [28]	individual	13 (p.K642E)	E, ST, SI, R	NS		yes	Vitiligo				
Kleinbaum et al. [29]	family	11 (c.D579del)	ST, SI, C	S	yes	yes	Hyperpigmentation, nevi				
Woźniak et al. [30]	family	11 (p.Q575_577delinsH)	R	S, M	yes				Constipation		
Thalheimer et al. [31]	family	17 (p. N822Y)	ST, SI, A, R	S	yes	yes					
Campbell et al. [32]	family	11 (NS^i^)	ST, SI, C	NS			Dysplastic nevi, lentigines, darkening of labia minora pudendi				
Veiga et al. [33]	family	17 (p.D820Y)	ST, SI, R	NS	yes						1 endometrial stromal sarcoma
Kuroda et al. [34]	family	11 (p. V559A)	ST, SI, C	S		yes	Hyperpigmentation of external genitalia and axilla				
Vilain et al. [35]	family	13 (p. K642E)	ST, SI	S^f^		yes	Hyperpigmentation (multiple nevi in the axillae and trunk and spontaneously resolving childhood facial hyperpigmentation) and hypopigmentation consistent with WS^j^ type 2		Dysphagia	Oesophagus	
Nakai et al. [36]	family	11 (p. Y553K)	ST, SI, C	NS		yes					
Wadt et al. [37]	family	13 (p.K642E)	ST, SI	NS	yes	yes					1 breast cancer
Speight et al. [38]	individual	9 (p.K509I)	SI	S				Mastocytosis			
Bachet et al. [13]	family	13 (p.K642E)	ST, SI, C, R	S	yes	yes	Lentigines and nevi				Multiple cutaneous angiolypomas in one individual
Bachet et al. [13]	family	13 (p.K642E)	ST, SI	S		yes					
Neuhann et al. [39]	family	11 (p.L576P)	ST, SI, C	S		yes	Multiple lentigines on face, neck, chest, back, axillae, legs		Achalasia, dysphagia		
Yamanoi et al. [40]	individual	13 (p.K642T)	ST, SI	S		yes			Dyshpagia		
Adela Avila et al. [41]	family	11 (p.V559A)	SI	S			Diffuse melanosis, generalized lentiginosis, palmar crease hyperpigmentation		Dysphagia		
Jones et al. [42]	family	11 (p.D579del)	ST, SI	S							
Jones et al. [42]	family	11 (p.D579del)	ST, SI	S							
Bamba et al. [43]	family	11 (p.V560del)	ST, SI	S							
Forde et al. [44]	family	11 (p.D579del)	ST, SI	NS			Skin hyperpigmentation		Dysphagia		

^a^ *E* esophagus, *ST* stomach, *SI* small intestine, *ICV* ileocecal valve, *A* appendix, *C* colon, *R* rectum, *NS* not specified

^b^ *S* spindle cell, *E* epithelioid, *M* mixed spindle cell and epithelioid, *NS* not specified

^c^ M, GIST metastases

^d^ Diffuse Intersitial cell of Cajal hyperplasia

^e^ inferred from the mention of intestinal obstruction

^f^ inferred from published microphotographs

^g^ not specified in the referred paper; inferred from the diagnosis of “neurofibromatosis” previously made in several of the family members

^h^ café-au-lait macules were reported in one of the individuals originally thought to have neurofibromatosis; this could have influenced the term used

^i^ NS, not specified

^j^ Waardenburg syndrome

As shown in Table 1, other features of germline *KIT* mutants include: diffuse ICC hyperplasia (ICCH) (with related peristalsis disturbances), skin pigmentation alterations and mast-cell disorders. Sporadically, melanoma, non-GIST stromal tumors and breast cancer have been signaled. *KIT* mutations could be implied in the arousal of the former [49]; the latter two are likely incidental. Sometimes, non-GIST signs are the first reason for patients to seek medical help [15, 32, 35, 41, 44].

Skin pigmentation defects mostly occur in excess; nevertheless, hypopigmentation has been reported [28, 35]. Of note, lentigines and vitiligo can coexist [20]. Finally, pigmentation alterations consistent with Waardenburg syndrome type 2 have also been reported in the absence of pathognomonic mutations, candidating the detected *KIT* variant among the possible causes of this disease [35].

The role of *KIT* in ICC development and gut motility regulation, and in the development and neoplastic transformation of melanocytes and mast cells [49], explains some of the germline *KIT*-mutants’ features. Conversely, the variable manifestations of these signs have not been satisfactorily justified. In particular, the suggested association between dysphagia and *KIT* TK II domain mutations and the inability of *KIT* TK I domain mutations to affect skin pigmentation [11, 16] proved wrong [13, 28, 35]. Acute myeloid leukemia, seminoma/dysgerminoma and sinonasal NK/T-cell lymphoma, neoplasms related to *KIT* mutations (often in exon 17) [49], at the best of my knowledge have never been described in germline *KIT*-mutants (curiously, some familial germ-cell tumors revealed somatic *KIT* mutations [59]). K559I and D816V *KIT* mutations, found in GISTs, can cause familial mastocytosis without detectable GISTs [38, 60–63]. Thus, genotype-phenotype correlation appears loose [39].

*KIT* mutations in sporadic GISTs cluster in exons 11, 9, 13, 17 and 8, with a frequency of ~65 %, ~8 %, ~1 %, ~1 % and < <1 %, respectively [2, 64]. In germline mutants, however, the *KIT* mutational spectrum differs (Table 1). In fact, focusing each single GIST oncogenic mutation arousal (counting each *KIT*-mutant kindred as one): 1) mutations involve exon 9 in 1/37 (3 %) and exon 17 in 4/37 (11 %) cases; 2) among exon-11 mutations, substitutions (61 %) prevail over deletions and insertion/deletions (29 %) (reversing the 31 %/60 % proportion found in sporadic GISTs); 3) *KIT* exon-11 mutations appear enriched in p.V559A, p.W557R and p.L576P substitutions (48 %, versus 10 % of sporadic GISTs) [58]. These differences suggest a selection of favorable genotypes in germline mutants, possibly less life-threatening, because: 1) exon-9 mutations proved aggressive in imatinib-naive GISTs; 2) exon-17 mutations appear more favorable than exon-11 ones [65]; 3) 5’-end exon-11 mutations, especially deletions, are likely biologically severe [30]; 4) sporadic GISTs with exon-11 substitutions or duplications revealed smaller than those with exon-11 deletions, and the former featured lower mitotic rates, supporting a lower biological impact of exon-11 substitutions; 5) GISTs bearing p.V559A, p.W557R and p.L576P mutations tend to feature better relapse-free survivals [58].

The first step of GIST tumoral progression in germline *KIT*-mutants is ICCH. “ICCH” and “micro-GIST” are terms applied to a variety of microscopic/tiny CD117+ cell lesions. Despite gross dimensional criteria have been proposed for separating them [66], a distinction between diffuse (ICCH) and nodular/focal (micro-GISTs) lesions prevails [2, 50]. Thus defined, ICCH is a non-neoplastic polyclonal lesion [10], constituting the only known GIST precursor. Subsequent events leading to overt tumors follow those found in sporadic GISTs: chromosomes 14 and 22 deletions [20], and loss of heterozigosity (LOH) involving the *KIT* wild-type (WT) allele at progression to malignancy [29]. The oncological impact of individual syndromic *KIT*-mutant GISTs is estimated using the parameters adopted for their sporadic counterparts [67]. Besides, germline *KIT*-mutant subjects may suffer frequent/severe gut occlusion/hemorrage, due to the tumor numerousness.

*KIT* mutations alter KIT structure simulating SCF-binding induced activation (if in exons 8 and 9, coding for the extracellular, ligand-binding domain), or allowing the kinase activation loop to switch to activation (exon 11, juxtamembrane regulatory domain), or directly imparting an active conformation to TK domains (exons 13 and 17, intracellular ATP-binding region and activation loop, respectively). This explains the differences in imatinib sensitivity depending on the *KIT*-mutant exon [50], either germline or not. Relatively better results are achieved when mutations occur “upstream” to the imatinib targeted site (i.e. in *KIT* exons 8, 9 and 11; for the rare exon 8 mutations evidences are limited [22, 64]), while the highest resistance rates are found in exon-13 and 17 mutations [50].

Table 1 details the features of the published germline *KIT* mutant kindreds/individuals manifesting GISTs.

#### *PDGFRA*-mutant syndrome

PDGFRA is a type III TKR whose gene is mapped to 4q12 [51], probably sharing with *KIT* a common ancestor [68]. PDGFRA is physiologically activated through binding to all PDGFs except PDGF-DD [69], triggering the same pathways elicited by KIT [50] (Fig. 1), albeit differentially activating PI3K/AKT/mTOR over RAS/MAPK [70]. Coherently, activating mutations of *PDGFRA* and *KIT* can raise similar tumors and are usually mutually exclusive.

At the best of my knowledge, germline *PDGFRA*-mutant GISTs have been signaled in two kindreds and in an individual without familial history [45–48]. A *PDGFRA*-mutant family bearing intestinal tumors defined as GISTs, but lacking GIST hallmarks except *PDGFRA* status, has also been reported [71]; these tumors are probably fibrous tumors, possible variants of another GI *PDGFRA*-driven tumor: inflammatory fibroid polyp (IFP) [48]. Moreover, a germline *PDGFRA*-mutant individual bearing multiple IFPs (and a likely *PDGFRA*-unrelated GIST hosting a somatic *KIT* mutation) has been reported very recently [72]; this individual shares both germline defect and geographical origin with one of the above mentioned kindreds [48], and is therefore probably related to it. Finally, three families featuring multiple IFPs have been described without *PDGFRA* genotyping [73–76]. *PDGFRA*-mutant syndrome is the term proposed for defining this clinical spectrum [48], formerly termed “intestinal neurofibromatosis/neurofibromatosis 3b” [71, 77, 78].

First diagnoses of IFP (including tumors misdiagnosed as neurofibromas) tend to precede those of GIST (means 40.6 and 48.1 years, respectively, *p* = 0.12 -Mann–Whitney *U* Test-) in *PDGFRA*-mutant syndrome, suggesting a faster IFP tumorigenesis (of note, IFP and GIST are unrelated lesions) [45, 47, 48, 71, 72, 78].

Although *PDGFRA*-mutant syndrome features an autosomal dominant pattern of inheritance with high penetrance [48], the sex distribution of GI tumors is apparently unbalanced, with a 4:11 male-to-female ratio (7/15 considering also pathologically undiagnosed GI tumors). Affected females outnumber males (11:1) also in ungenotyped kindreds featuring familial IFPs or “intestinal neurofibromatosis/NF3b” (12:2 including suspected GI tumors) [45–48, 71–78]. However, females prevail (14:5) also among the 19 ascertained *PDGFRA*-mutants, compensating the corresponding 10:4 female-to-male tumor distribution (coherently, Fisher exact test, one-tailed, proved not significant: *p* = 0.60).

*PDGFRA*-mutant GISTs (germline or not) are mostly at-least-in-part epithelioid, and gastric [48, 50]. The latter location appears so far exclusive in germline-mutant examples, as the only extra-gastric GIST reported in this genetic setting bore a concomitant somatic *KIT* mutation, and likely hinged exclusively on it [72]. *PDGFRA*-mutant GISTs express CD117 (less frequently than *KIT*-mutant GISTs and often weakly/patchy) and DOG1.

*PDGFRA*-mutant syndrome may also feature IFPs (including GI fibrous tumors), GI lipomas or large hands (Table 2). Diffuse ICCH has never been described in germline *PDGFRA* mutants; the reported “focal ICCs” [45] rather fits micro-GIST. These findings support *PDGFRA*-mutant and *KIT*-mutant GISTs as distinct entities. Accordingly, GI motility disturbance, frequently accompanying ICCH, is not typical of *PDGFRA*-mutant syndrome. Although the *PDGFRA* role in GIST and IFP pathogenesis [79] justifies the presence of these two tumor types in *PDGFRA*-mutant syndrome, the occurrence of GI lipomas (sporadic GI lipomas revealed *PDGFRA* WT [80]) and large hands, and the variability of the observed phenotypic assortment are presently unexplained.

**Table 2:** Kindreds and individuals affected by *PDGFRA*-mutant syndrome

Reference	Type of report (family vs. single individual)	Mutant exon (mutation)	GIST			Associated signs				
				Site^a^	Histology^b^	IFP^c^	GI fibrous tumors^d^	GI lipomas	Large hands	Other manifestations
Chompret et al. [45]	family	18 (p.D846Y)	yes	ST	M (mainly E)				yes	
de Raedt et al. [71]; Heimann et al. [78]	family	12 (p.Y555C)					yes^e^		yes	Broad wrists, glaucoma
Pasini et al.[46]; Carney and Stratakis [47]	individual	12 (p.V561D)	yes	ST	M (mainly E)		yes	yes		
Ricci et al. [48]	family	14 (p.P653L)	yes	ST	E, M (mainly E)	yes	yes	yes		
Ricci et al. [72]	Individual (mother and grandmother suffered a gut occlusion) (likely related to the above mentioned kindred [48])	14 (p.P653L)	yes (bearing a concomitant somatic *KIT* mutation)	SI	S	yes				

^a^: *SI* small intestine, *ST* stomach

^b^: *E* epithelioid, *M* mixed spindle cell and epithelioid; *S* spindle cell

^c^: Inflammatory fibroid polyp

^d^: Fibrous tumor is likely a variant of IFP [48]

^e^: When a germline *PDGFRA* mutation was found in the referred kindred, these tumors were considered GISTs since GISTs were the only *PDGFRA*-mutant GI tumors known at that time

GIST *PDGFRA* mutations cluster in exons 12 (juxtamembrane regulatory domain), 14 and 18 (TK domains -ATP binding region and activation loop, respectively-). Mutations in all of these exons are represented in *PDGFRA*-mutant syndrome (Table 2). Two of them (involving exon 12) are relatively indolent, one at intermediate risk (exon 14) and another probably at high risk (exon 18-non-p.D842V) [65]. Besides, *PDGFRA* exon-14 mutations proved prognostically favorable in another work [81]. Similarly to *KIT*-mutant GISTs (counting each kindred as one, and considering the recently reported p.P653L individual [72] as a member of a previously published *PDGFRA*-mutant family [48] given the identity of both genetic defect and geographical origin), with the caveats of the limited sample, *PDGFRA* mutation distribution differs in germline mutations with respect to sporadic GISTs, with exon 18 involved in 25 % (Table 2) and 82 % [65] of cases, respectively. Germline-mutants appear enriched in presumably low-biological-impact mutations [65], with low molecular risk exon-12 ones found in 50 % of cases.

Germline *PDGFRA*-mutant GISTs feature 14q LOH [46], similar to sporadic, germline *KIT*-mutant and NF1-associated ones.

In *PDGFRA*-mutant kindreds, small bowel occlusions due to IFP can be life-threatening. Of note, only one individual died of malignancy possibly related to GIST described as “gastric cancer” in the absence of pathological records [48], coherently with the relative indolence of *PDGFRA*-mutant GISTs [82]. In need of treatment, however, TKI use must be pondered, evaluating the specific mutation present. In fact, although never reported in germline-mutants, p.D842V, the most common *PDGFRA* mutation of sporadic GISTs, confers resistance to both imatinib and the second-line TKI sunitinib, justifying alternative approaches using dasatinib and crenolanib [83–85].

Table 2 details the features of published *PDGFRA*-mutants syndrome cases.

#### NF1-associated GISTs

Neurofibromin, encoded by *NF1* gene on 17q11.2 [51], accelerates the conversion from active GTP-bound to inactive GDP-bound RAS. NF1 inactivation stimulates MAPK cascade through increasing RAS activity [50], promoting tumorigenesis (Fig. 1).

NF1 is relatively common, with a ~1:3,000 birth incidence and a 1:4–5,000 prevalence [86]. NF1 transmission is autosomal dominant with complete penetrance and variable expression. ~50 % of NF1 lack familial history due to the high human *NF1* mutation rate. In NF1, one *NF1* allele is germline-mutant, the other displays tumoral somatic inactivation.

National Institutes of Health NF1 diagnostic criteria consist of ≥2 among: ≥6 café-au-lait macules >5 mm or >15 mm in pre-pubertal or post-pubertal subjects, respectively; ≥2 neurofibromas (one if plexiform); axillary/inguinal freckles; optic glioma; ≥2 iris hamartomas; bony dysplasia; and a NF1-affected first-degree relative [87]. 0.1-6 % of NF1 feature paragangliomas [88].

Clues of a possible association between NF1 and GIST have been known for decades [89]. As differential diagnosis between neurofibroma (NF) and GIST was made reliable [90], this association became evident. GISTs are the most frequent GI NF1 manifestation with a 7 % prevalence in NF1 patients, increasing to 25 % at autopsy. NF1 is >45-fold overrepresented among GIST patients. Coherently, *NF1*-mutated GISTs account for ~1,5 % of all GISTs. Average age-at-diagnosis of NF1-associated GISTs is ~49 years [1, 86, 91, 92].

NF1-associated GISTs are often multiple and small intestinal, with possible gastric exceptions (NF1 frequency is 6 %, 4 % and 0.06 % among patients with duodenal, jejunal/ileal and gastric GISTs, respectively); singly considered, they do not differ from sporadic intestinal GISTs, mostly featuring spindle cells, collagen globules (skeinoid fibers) and CD117 positivity [91]. Other NF1 GI lesions include: ICCH, with related GI motility disorders, and neuroendocrine tumors (especially periampullary somatostatinomas) [86]. The awareness of these GI signs can help to avoid diagnostic omissions caused by the variable clinical presentation and frequent non-familial form of NF1 (the lack of *NF1* mutational hotspots makes genotyping an unpractical diagnostic tool) [86].

The discovery of *NF1* second-hit in NF1 GISTs, already revealed peculiar because of their usually WT *KIT*/*PDGFRA* [91, 93], disclosed their pathogenesis and molecular link with NF1 [94]. *KIT*/*PDGFRA* mutations in NF1-associated GISTs are nevertheless possible, although probably incidental [95], globally accounting for ~8 % of cases [96].

NF1 GIST tumoral progression resembles that of germline *KIT* mutants, featuring preneoplastic ICCH followed by 14q and 22q LOH [97].

Although mostly indolent, ≤15-20 % of NF1 GISTs behave aggressively [91, 93]. NF1 GISTs are poorly responsive to imatinib, since NF1 trigger occurs downstream to imatinib targets KIT/PDGFRA; coherently, NF1 GISTs should not be treated with adjuvant imatinib, whatever their risk [98–100].

#### Syndromic SDH-deficient GISTs

SDH is a 4-subunit (A/B/C/D) Krebs cycle enzymatic complex, located in the inner mitochondrial membrane, but encoded by chromosomal DNA. *SDHA*/*B*/*C*/*D* (collectively, *SDHx*) are mapped to 5p15.33, 1p36.13, 1q23.3 and 11q23.1, respectively [51]. Whatever subunit is damaged, the entire complex is hampered, impairing succinate-to-fumarate conversion; succinate accumulation inhibits prolyl-hydroxylase, decreasing the hydroxylation of hypoxia-inducible factor (HIF)-1α (HIF-1α) and its consequent degradation; HIF heterodimers can thus translocate into the nucleus initiating tumorigenic transcription. Furthermore, succinate accumulation inhibits TET DNA-hydroxylases, compromising the 5-methylcytosine conversion to 5-hydroxymethylcytosine, required for DNA demethylation; coherently, *SDHx*-deficient GISTs feature pervasive DNA hypermethylation, likely implied in oncogenesis [101–105] (Fig. 1).

SDH-deficiency characterizes the largest *KIT/PDGFRA*-WT GIST subgroup (accounting for ~5 % of GISTs) [1]. SDH deficiency is also a feature of paragangliomas, renal cell carcinomas and pituitary adenomas [106, 107]. SDH-deficient GISTs arise either in germline *SDHx*-mutant and/or manifest syndromic settings or not [105, 108]. Thus, not surprisingly, in case of constitutive predisposition to SHD-deficiency, GISTs can associate with paragangliomas producing two syndromes: Carney’s triad (CT) and Carney-Stratakis Syndrome (CSS), both affecting young people (mean ages-at-diagnosis 22 and 19 years −22 and 24 years concerning GIST only-, respectively) [109–111]. CT and CSS are separated by the presence of pulmonary chondromas and of a striking female predilection in the former, and of an autosomal-dominant inheritance pattern in the latter, with incomplete penetrance [111]. Additionally, CT may feature esophageal leiomyoma and adrenal cortical adenoma [110]. SDH-deficient GISTs are restricted to stomach (especially antrum) and show a multinodular pattern (referred to as “plexiform” by pathologists) [103, 110].

≤50 % and ≤10 % of SDH-deficient GISTs manifest lymph-vascular invasion and lymph node metastases, respectively [112], explaining the frequent relapses despite apparently radical surgery.

SDH-deficient GIST are mostly at-least-in-part epithelioid, and express DOG1, CD34 (≥75 % of cases) and CD117 (strongly/diffusely, unlike *PDGFRA*-driven GISTs) [103, 110]. They are peculiarly SDHB-, whatever the damaged SDH subunit (unlike SDHA positivity, lost only in *SDHA*-mutations), and overexpress insulin-like growth factor 1 receptor (IGF1R) [103, 113–115].

Only micro GISTs have been found to precede overt SDH-deficient syndromic GIST [116]. ICCH is not a feature of CT [110], nor has ever been described in SDH deficiencies.

The basis of SDH impairment in CSS is mutational, with a typical second-hit mechanism involving *SDHB*/*C*/*D* [117, 118]. Significantly, germline *SDHA* mutations, described in patients bearing GISTs or paragangliomas and in paraganglioma/pituitary adenoma familial associations, are unreported in CSS, possibly due to their low penetrance [106, 108, 115, 118–125]. Of note, at the best of my knowledge, the only *SDHD*-mutant CSS with a reported pedigree hinged on a paternally inherited defective allele [126], coherently with the parent-of-origin effect of *SDHD*-depending hereditary paraganglioma syndrome PGL1, simulating maternal imprinting. However, *SDHD* lacks physical imprinting and PGL1 is exceptionally maternally transmitted, invoking the involvement of a second, paternally imprinted, tumor suppressor gene (TSG) (possibly *H19*) located on 11p15, i.e. the same chromosome of *SDHD*. Both WT *SDHD* and functional 11p15 TSG can thus be inactivated by non-disjunctional loss of the maternal chromosome 11, justifying the paternal disease transmission. More complex events, such as mitotic recombination of 11p15 TSG followed by loss of the paternal chromosome, would explain the exceptional maternal transmission [127–129].

Unlike CSS, CT tumorigenesis depends on epigenetic tumoral SDHC inactivation through *SDHC* hypermethylation [130]. This phenomenon, distinct from the global DNA hypermethylation of SDH-deficient GISTs as a whole, is common to most SDH-deficient, *SDHx*-WT GISTs, irrespective of CT presence; however, “non-CT” cases could be formes frustes of CT, as suggested by the mosaic constitutional *SDHC* promoter hypermethylation, implying a risk for metachronous paraganglioma/pulmonary chondroma [105].

Another oncogenic stimulus of SDH deficient GISTs is the hyperactivation of the IGF1R pathway, physiologically involved in cell survival/proliferation [113, 131].

≥50 % CT GISTs do not bear chromosomal imbalances; their rare LOHs preferentially involve 1p; 14q or 22q losses occur infrequently [132].

SDH-deficient GISTs often behave indolently, even in case of metastases, probably due to the metabolic disadvantage caused by SDH deficiency. They can nevertheless be aggressive (the SDH-deficient GIST overall mortality approaches 15 %). Of note, current risk classifications do not fit SDH-deficient GISTs [110, 112].

TKI imatinib and sunitinib proved ineffective or only partially effective, respectively, in SDH-deficient GISTs, coherently with SHD deficiency constituting a pathway independent of KIT/PDGFRA. Therefore, in the absence of guidelines specific for these tumors, TKI treatment is suggested for progressive disease but not after complete surgical resection [98–100, 112]. Inhibitors of IGF1R are being evaluated: testing of linsitinib in adult and pediatric WT GISTs (enriched in SDH-deficient GISTs), although without evidence of RECIST response, indicates a 45 % clinical benefit/52 % progression-free survival at 9 months [133]. When compared to *KIT*-mutant GISTs, a higher fraction of *KIT*/*PDGFRA*-WT (and *PDGFRA*-mutant) GISTs displayed activation of the mTOR pathway [70]; Therefore, CT/CSS GISTs can potentially benefit of PI3K/AKT/mTOR inhibitors [131]. Regorafenib, nilotinib, sorafenib and heat-shock protein inhibitors are other drugs potentially usable; finally, targeting of HIF-1α, α-ketoglutarate, and demethylating agents such as decitabine are theoretically attractive approaches [104, 105, 134–136].

CSS should be readily distinguishable from CT based on the presence or not of pulmonary chondromas, germline *SDHB/C/D* mutations and inheritance; female predilection of CT is another helping feature [137]. However, the arousal of tumors in SDH-deficient syndromes, including CT and CSS, can span over dozens of years [112]. Thus, if the “missing” tumor of a CT is pulmonary chondroma, CT/CSS morphologic differential diagnosis is impossible. Under these circumstances, in the absence of familial history, genotyping becomes pivotal. But recent findings have eroded also this cornerstone, evidencing germline *SDHA*/*B*/*C* variants, at least part of which pathogenic, in 6/63 (9.5 %) CT patients “certified” by pulmonary chondromas. On top of that, unlike typical CT, these patients were equally distributed between sexes [138]. Additionally, three males diagnosed with CT (one with a lesion “consistent with pulmonary chondroma”) were subsequently found to bear germline *SDHx* mutations [105, 139]. Noticeably, CT diagnosis in these cases appears questionable, with genotype and sex distribution rather supporting CSS.

GISTs could be also considered an infrequent component of SDH-deficient paraganglioma syndromes PGL1/3/4/5 [140], depending on *SDHD*/*C*/*B*/*A* germline mutations, respectively [141]. A risk of WT *SDHx* allele loss lower in GIST precursor cells than in paraganglial ones could explain the relative GIST rarity [112]. Noticeably, associations between GIST and renal cell carcinoma, another SDH-deficient tumor reported in paraganglioma syndromes, has been found in germline *SDHA*/*B/C* mutants [141–144].

Perhaps CT, CSS and PGL1-5 will be reconsidered in the future as different aspects of a single SDH-deficient disease.

### Practical approach to GIST-predisposing syndromes

GIST-prone syndromes must be suspected in the presence of GISTs either multiple or associated with the peculiar manifestations previously described (which can raise suspicions even by themselves) (Table 3). Genotyping, if applicable, is confirmatory. NCCN guidelines suggest to investigate every *KIT/PDGFRA*-WT GIST patient for germline *SDHx* mutations [84]. Multiple GISTs sharing a phenotype not found in the germline favor a metastatic condition.

**Table 3:** Main features of GIST-predisposing syndromes

Syndrome		Trigger	Inheritance	Sex predilection	Average age at diagnosis (years)	GIST features			Other manifestations
						Site^a^	Morphology^b^	Immunohistochemistry	
*KIT*-mutant		germline *KIT* mutation	Autosomal dominant, high penetrance	None	48	SI, ST > C, R > E	S > M> > E	CD117+ DOG1+	Skin hyperpigmentation, mast cell disorders, ICCH^c^, dysphagia
*PDGFRA*-mutant		germline *PDGFRA* mutation	Autosomal dominant, high penetrance	None	48 (GIST), 41 (inflammatory fibroid polyp)	ST	E, M	CD117+/− DOG1+/−	Inflammatory fibroid polyps (including GI^d^ “fibrous tumors”), GI lipomas, large hands
Neurofibromatosis type 1		Germline *NF1* mutation + tumor 2nd hit in WT allele	Autosomal dominant, complete penetrance and variable expression	None	49	SI > ST	S > M	CD117+ DOG1+	Neurofibromas and other signs of Neurofibromatosis type 1, ICCH, dysphagia
SDH-deficient syndromes	CT^e^	epigenetic SDHC promoter hypermethylation in tumors	None^f^	F> > M	22 (either whatever tumor type or GIST)	ST	E > M, S; plexiform	CD117 + DOG1+	Paragangliomas/pheocromocytomas, pulmonary chondromas, esophageal leiomyoma, adrenal cortical adenoma
	CSS^g^	Germline *SDHB*, *C* or *D* mutation + tumor 2nd hit in WT allele	Autosomal dominant, incomplete penetrance. Parent-of-origin if *SDHD*-mutant	None	19 (whatever tumor type), 24 (GIST)	ST	E > M, S; plexiform	CD117 + DOG1+	Paragangliomas/pheocromocytomas

^a^ *E* esophagus, *ST* stomach, *SI* small intestine, *C* colon, *R* rectum

^b^ *S* spindle cell, *E* epithelioid, *M* mixed spindle cell and epithelioid

^c^ Diffuse Intersitial cell of Cajal hyperplasia

^d^ GI, gastrointestinal

^e^ Carney’s triad

^f^ Recently reported 6 germline *SDHx*-mutant cases, one of which with inherited paragangliomas

^g^ Carney-Stratakis syndrome

Comprehensive guidelines specific for the management of GIST-prone syndromes are lacking. Existing recommendations, dealing with adjuvant therapy of NF1-associated and SDH-deficient GISTs [99], have been treated in the pertaining chapters of this review.

Once a patient is diagnosed with a GIST-predisposing condition depending on a germline DNA defect, predictive genetic testing in family members should be considered. The latter is indicated for the highly penetrant germline *KIT*/*PDGFRA*/*SDHB*/*SDHD* mutations; conversely, the opportunity of genetic evaluation is controversial for the low penetrance *SDHC* and, especially, *SDHA* variants [141]. It is worth recalling the parent-of-origin effect of familial *SDHD* mutations, herein previously discussed.

Periodic computed-tomography-scan or ^18^F-FDG PET-computed-tomography have been suggested for the surveillance of syndromic GISTs [145]. Endoscopic ultrasound joined to fine needle tissue acquisition allowing histological assessment [146, 147] appear valid complements. Colonscopy is expectedly of limited utility in familial *KIT*-dependent GISTs [29] (mostly gastric/small intestinal) and useless in SDH-deficient ones (strictly stomach restricted), while can detect colonic/ileal IFPs in *PDGFRA*-mutant syndrome [48]. In case of SDH-deficient GISTs, chest X-ray is indicated to look for pulmonary chondromas (especially if *SDHx*-WT), and plasma/urine determination of metanephrines/catecholamines and PET-tracers ^68^Ga-DOTATATE, ^18^F-DOPA and ^18^F-FDA can be used for investigating paragangliomas [141].

Intracellular signaling machineries and gene expressions are common to homologous syndromic and sporadic GISTs [20]. Coherently, no differences in imatinib sensitivity exist between GISTs sharing the same genotype, no matters whether somatic or germline [98]. Thus, the approach to individual syndromic GISTs in need of treatment does not differ from that to their sporadic counterparts.

There is no evidence that GIST hereditary predispositions constitute an independent prognostic factor warranting a differential GIST treatment [145]. Nevertheless, the frequent multiplicity of GI tumors constitutes a problem peculiar to GIST-syndromes deserving a special attention, commonly causing acute complications such as hemorrage and, especially in germline *KIT*/*PDGFRA* mutations and NF1 (where the small bowel can be involved), occlusion/perforation which can be life-threatening [13, 43, 48]. Although surgery is frequently necessary as affected patients are often symptomatic [13], no agreement exists as to whether preventive therapy, either surgical or molecularly targeted, is indicated in asymptomatic subjects [145]. It has been suggested to postpone surgery based on the frequent indolence of germline-mutant GISTs [148]; however, the latter’s possible aggressiveness recommends to remove GISTs at presumable significant risk, based on size/site/genotype. Similarly, lesions at risk of occlusion/hemorrhage should be removed too. Surgery should focus on resecting the outstanding tumor(s) disregarding possible tiny nodules if numerous and involving extended areas, since their removal would sacrifice substantial portions of functional GI tissue without proven benefit in terms of tumor recurrence risk; this is particularly true in CT and CSS patients, given their frequently young age [109]. Preventive molecular therapy poses several problems. In fact, although theoretically attractive, it is presently not evidence-based. Prospective clinical trials will be hardly achieved, given the rarity of syndromic GISTs. Moreover, the risks of lifelong exposures to GIST-targeted drugs are presently unknown. Thus, active surveillance has been adopted after surgery [44]. Alternatively, a long term preventive treatment with TKI in patients bearing a sensitive mutation has been proposed [13]. A compromise has been adopted by employing half-dose imatinib in a *KIT* exon-11 germline mutant with multiple GISTs, obtaining marked tumor reductions after one-year [43]. Interestingly, imatinib therapy has been reported to reduce cutaneous melanosis in germline *KIT*-mutants [32, 41].

Exceptionally, GISTs in syndromic contexts can reveal superimposed triggers [96, 149]. Anomalous GIST site and/or morphology with respect to a given hosting syndrome can help in suspecting these “divergent” GISTs, whose pathogenesis can hinge mainly or even exclusively upon the “extra-syndromic” molecular defect, with relevant clinical implications [72]. In any event, to be on the safe side it is advisable to fully genotype whatever apparently syndromic GIST to be molecularly treated, independently of its morphology and site.

## Conclusions

The correct approach to syndromic GISTs results from the integration between congruent diagnostic strategies, including familial screening, and treatment of individual tumors and background syndromic manifestations. Protean signs prompt to undertake complex choices involving the treatment of symptomatic lesions and the prevention of future complications. Specific comprehensive guidelines are lacking, primarily due to the rarity of syndromic GISTs. However, these diseases have been the subject of an increasing number of publications, resulting in a conspicuous amount of data with relevant clinical implications. The latter allowed the draft of the present review, which hopefully will help physicians facing GIST-prone syndromes, waiting for the development of dedicated guidelines.

## Abbreviations

CSS, Carney-Stratakis syndrome; CT, Carney’s triad; GIST, gastrointestinal stromal tumor; ICCH, interstitial cell of Cajal hyperplasia; IFP, inflammatory fibroid polyp; NF1, neurofibromatosis type 1; PDGF, platelet-derived growth factor; SCF, stem cell factor; SDH, succinate dehydrogenase
